# Focal therapy for prostate cancer – index lesion treatment vs. hemiablation. A matter of definition

**DOI:** 10.1590/S1677-5538.IBJU.2019.05.02

**Published:** 2019-01-29

**Authors:** Armando Stabile, Marco Moschini, Francesco Montorsi, Xavier Cathelineau, Rafael Sanchez-Salas

**Affiliations:** 1 Urological Research Institute Vita-Salute San Raffaele University IRCCS San Raffaele Scientific Institute Milan Italy Department of Urology and Division of Experimental Oncology, URI, Urological Research Institute, Vita-Salute San Raffaele University, IRCCS San Raffaele Scientific Institute, Milan, Italy;; 2 Klinik für Urologie Luzerner Kantonsspital Lucerne Switzerland Klinik für Urologie, Luzerner Kantonsspital, Lucerne, Switzerland;; 3 Department of Urology Institut Mutualiste Montsouris Université Paris Descartes Paris France Department of Urology, Institut Mutualiste Montsouris and Université Paris Descartes, Paris, France

Current standard of care for localized prostate cancer (PCa) include active surveillance and radical therapy. Tissue-sparing approaches such as focal therapy (FT) has recently emerged to cover that middle ground between active surveillance and whole gland therapies in order to provide cancer control while reducing morbidities and side-effects. Evidence from a systematic review including thirty-seven studies reporting on 3230 patients receiving FT through different energy sources reported a rate of significant disease (csPCa) at follow-up biopsy ranging between 0% and 13% within a median follow-up ranging from 4 to 61 months. Leak-free continence and potency rate were 83.3-100% and 81.5-100%, respectively (1). The largest series (n=1032) of men receiving FT using high-intensity focused ultrasound (HIFU) reported a freedom from csPCa at follow-up biopsy of 54% at 8 years from treatment, with 46% of men being free from any additional treatment at the same time point (2). Yap et al, reported functional results of 180 patients submitted to tissue-preserving therapy, describing no significant changes in International Index of Erectile Function (IIEF) from preoperative to 12-months values (3). Nonetheless, due to the lack of consistent and long-term results and the presence of several difficulties in providing standardized treatment and follow-up strategies, FT has been proposed by the European Association of Urology as an investigational modality (4). This statement raised several arguments supporting the potential useful role of FT (5,6), underlining that other innovative therapeutic strategies has been recommended in the past before the availability of long-term outcomes or data from randomized controlled trials (e.g partial nephrectomy for kidney cancer, tumorectomy for breast carcinoma). The trade-offs that patients are willing to make in terms of ratio between rate of treatment failure and rate of side-effects is key. We must avoid to deny our patients a potential further therapeutic strategy, that many would accept as an alternative.

The most controversial topic against FT is the multifocal nature of PCa. Indeed, PCa is multifocal in up to 75% of cases which is in contrast with the possibility of achieving cancer control with a focal treatment of the prostate gland. Nonetheless, in the last few years, several efforts have been made in order to demonstrate the “index lesion theory” according to which the largest and most aggressive tumour focus within the prostate drives the natural history of the disease (7). Given the fact that in up to 25% of cases PCa is a true unifocal disease, there are some evidences demonstrating that PCa metastasis have often a monoclonal origin, and that in a non-negligible proportion of cases these metastatic cells derive from the index lesion (IL) (8,9). In a recent survey study including 425 urologist showed as only 45% of the participants believed in the “index lesion theory”, with a higher incidence of believer coming from academic centres (10). Even though this topic remains one of the most controversial in the field of PCa biological nature, evidences supporting the possibility to achieve acceptable cancer control through FT, at least at mid-term follow-up, are increasing (2).

During the treatment planning process of FT is therefore considered pivotal aiming at accurately covering the so called IL, regardless of the energy used.

The introduction of multiparametric MRI of the prostate together with the possibility to perform targeted biopsies, has provided a cornerstone tool in the patient selection process when talking about FT, helping to identify the IL and rule out the presence of surrounding csPCa foci (11). Although the combination of MRI and targeted biopsies represent the gold standard for FT patient selection, the diagnostic accuracy of this strategy is still unclear. Nassiri et al. demonstrated as eligibility for FT was confirmed in 75% of men considered eligible with the use of MRI targeted biopsies (12). On the other hand Jonson et al. showed as, using the same diagnostic technique, up to 48% men would have been incorrectly identified as having unilateral PCa (13). Being that said, the use of MRI and targeted biopsies is suggested as the probably the most efficient method to provide an acceptable mapping of the prostatic gland.

Identifying the IL and achieving a good treatment coverage of it during FT is crucial. Based on the characteristic of the disease, particularly the volume and the extension within the prostate, different treatment strategies, with different energy sources, have been proposed: I) “index lesion ablation or focal-ablation” when the treatment is limited to the IL, plus safety margins; II) “hemi-ablation” consists in the treatment of the half of the prostate containing the tumour; III) “sub-total ablations” when the ablation volume is greater than half the prostate, for example hokey-stick ablation (14,15). Each treatment should include safety margins around the area containing the tumour, which usually account for 5mm up to 9mm of normal tissue (2,16). Nowadays the most common used FT strategy is represented by the hemi-ablation (1). Evidences comparing the efficacy of focal- vs hemi-ablation are so far scarce. To the best of our knowledge, Stabile et al. reported the first comparison between focal- and hemi-ablation in terms of the rate of any additional treatment after FU in a population of over one-thousand men receiving primary FT for PCa using high-intensity focused ultrasound (HIFU), reporting no differences between the two strategies (2). Nonetheless, regardless the pre-operative treatment plan, in an intra-operative setting is often challenging discerning between focal- and hemi-ablation, considering safety margins and the different prostate volumes. The difference between focal- and hemi-ablation has been maintained over the years in order to better describe the amount of prostatic tissue spared during FT. Indeed, the more extensive the treatment, the more likely the functional outcomes will get close to those of whole gland therapies (i.e. radical prostatectomy, radiotherapy). Therefore it becomes of great importance, also considering patient counselling, the possibility to give an idea regarding the extension of FT treatment. However, given the extreme variability of IL and prostates characteristics, concerning volume, extension and shape, pushing FT treatments into the aforementioned definitions (i.e. focal-, hemi- and subtotal-ablation) might be not completely accurate for patients counselling as well as for outcomes reporting. Focal therapy represents indeed a continuum of treatment extensions and strategies which is adapted according to each clinical case. Sivaraman et al. two years ago, proposed an “À la carte” approach when delivering FT, that was then officially recognized and validated by the European Society of Urotechnology (ESUT) (17,18). Particularly the authors showed the possibility of choosing different energy modalities according to the intraprostatic cancer location, more specifically using HIFU for posterior tumours and preferring cryotherapy for anterior tumours (17). With the growing use of MRI and targeted biopsies and its introduction in the FT patients selection pathway, the location and extension of the IL has become clear and quite reliable as well as exploitable by the urologist. The concept of tailoring FT treatment on cancer characteristics, particularly the intraprostatic extension, is routinely performed, for every type of FT energy sources. For these reasons we might assert that specifying the difference between focal- and hemi-ablation could be, in the next future, considered obsolete. Indeed, there is a call for further improvements in the field of intraprostatic mapping and refinement of FT devices and interest is growing around the study of prostate tumour microenvironment and its modulation after specific treatments (19). Further developments in the possibility to better define the prostatic area to treat and to identify prostatic tissue eventually treated will make a step forward towards the standardization of FT treatments and consistency of provided oncologic and functional results.

According the results of this experimental study during a partial nephrectomy, the en bloc clamping for warm ischemia should be favored over only the renal artery clamping to minimize renal injury after partial nephrectomies, but more studies will be necessary in the future to confirm these results.
